# French Canadian cross-cultural adaptation of CAPTSure©, an index for the Clinical Assessment of Post-Thrombotic Syndrome in children

**DOI:** 10.1186/s41687-023-00622-7

**Published:** 2023-08-18

**Authors:** Marie-Claude Pelland-Marcotte, Angelika Stavrakoukas, Gina Wong, Raoul Santiago, Laura Avila

**Affiliations:** 1 Division of Pediatric Hematology-Oncology, Centre Hospitalier Universitaire de Québec – Centre Mère-Enfant Soleil, Quebec City, QC Canada; 2 Centre de recherche du Centre Hospitalier Universitaire de Québec, Quebec City, QC Canada; 3 Division of Haematology/Oncology, The Hospital for Sick Children, Toronto, ON Canada; 4 Child Health Evaluative Sciences, Research Institute, The Hospital for Sick Children, Toronto, ON Canada; 5 Dalla Lana School of Public Health, University of Toronto, Toronto, ON Canada

**Keywords:** Children, Thrombosis, Post-thrombotic syndrome, Cross-cultural adaptation

## Abstract

**Purpose:**

Post-thrombotic syndrome (PTS) is the most common complication of deep venous thrombosis (DVT). The index for the Clinical Assessment of Post-Thrombotic Syndrome in children (CAPTSure©) is a clinical tool for the diagnosis and severity rating of PTS in pediatric patients. The purpose of this study was to translate and adapt CAPTSure© for French-speaking patients.

**Methods:**

We conducted a cross-sectional study to perform linguistic and cultural adaptation of CAPTSure©, using a rigorous translation process followed by cognitive debriefings in twenty French-speaking pediatric patients aged up to 18 years old with a history of upper or lower extremity DVT at least 6 months prior.

**Results:**

Forward and backward translations were used to produce a pre-final French version of CAPTSure©, followed by cognitive debriefings in twenty participants (median age: 11.5 years, 55% male, median CAPTSure© score: 26). The participants felt that the questionnaire was thorough, with an adequate length. Eight out of fourteen (57%) items in the LE questionnaire and 7/12 (58%) of the items in the UE questionnaire were modified following participants’ and a multidisciplinary expert committee’s input, leading to the final French version of CAPTSure©.

**Conclusions:**

CAPTSure© was successfully adapted for French-speaking pediatric patients. This will ease the diagnosis and severity rating of PTS in children in clinical practice and allow international research collaborations for additional non-English-speaking patients.

**Supplementary Information:**

The online version contains supplementary material available at 10.1186/s41687-023-00622-7.

## Background

The incidence of venous thromboembolism (VTE) is rapidly increasing amongst all pediatric age groups [1]. Post-thrombotic syndrome (PTS) is the most common complication of deep venous thrombosis (DVT), touching approximately one out of two to four affected children [2]. PTS, defined as chronic venous symptoms and signs secondary to DVT [3], can impact patient functioning, happiness, and health-related quality of life [4].

The index for the Clinical Assessment of Post-Thrombotic Syndrome in children (CAPTSure©) is a clinical tool developed to diagnose and rate the severity of PTS in pediatric patients. An international group of thrombosis experts and patients and their families were involved in eliciting signs and symptoms to be included [5]. Items reduction and weighting followed a modified Delphi process so that scoring reflects the relative importance of signs and symptoms to the healthcare providers, patients and families [6], It has well-documented validity and reliability and has a small measurement error even when performed by non-hematologists [7].

To implement its use in clinical practice for French-speaking patients and to enable their participation in international multicenter trials, comparable versions of CAPTSure© are needed in French. This requires both linguistic translation and cultural adaptation, followed by an evaluation process to ensure adequacy in its new setting and comparability with the original tool [8]. The purpose of this study was to translate and adapt CAPTSure© for French-Canadian patients.

## Methods

**Fig. 1:**
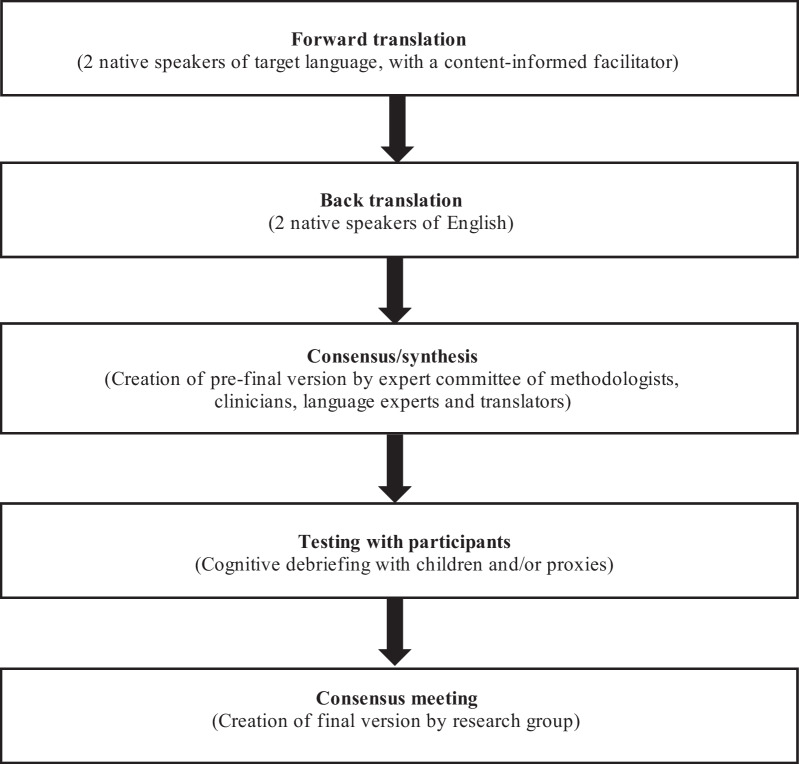
Review of the cross-cultural adaptation process

We performed a cross-sectional study, following previously published approaches for cross-cultural adaptation of measurement instruments [9, 10], with adaptations suggested by Price and colleagues [8]. In brief, these adaptations allow a smaller sample size due to the relative rarity of pediatric diseases and give pivotal importance to a bilingual clinical expert during translation to keep the linguistic style appropriate for children [8]. This process followed five steps: (1) forward translation; (2) backward translation; (3) review of source and translated versions by a multidisciplinary team, (4) pre-testing for equivalence using cognitive debriefing, and (5) consensus meeting (Fig. 1). The multidisciplinary team included members of the research group who developed CAPTSure© in its original version, including a clinician-scientist with content expertise in pediatric thrombosis and methodological expertise in the development of patient-reported outcomes, research personnel, translators from a Canadian commercial firm including a senior translator with experience in questionnaires, as well as one French-speaking clinician-scientist with expertise in pediatric thrombosis (Additional file 1: Table S1). Ethical approval for the study was obtained from the Research Ethics Boards of the Centre Hospitalier Universitaire (CHU) de Québec (approval number: #2021-5558).

### CAPTSure©

CAPTSure© comprises two sub-indices, for upper (UE) and lower extremity (LE). During the development of CAPTSure©, it was anticipated that clinical manifestations of PTS could differ between the upper and lower extremity, in view of physiological differences between the two vascular territories. Therefore, two sub-indices for the UE and LE were developed separately [5]. Both sub-indices encompass a “Symptoms component” and the “Clinician assessment component”, collecting symptoms and physical exam findings, respectively. The final score ranges between 0 and 100 points, with higher scoring indicating higher PTS severity; a score of 0–10 points indicates no PTS, a score of 11–30 points indicates mild PTS, and a score of ≥ 31 points indicates moderate to severe PTS [4]. Training videos were developed for its administration [11]. The questionnaire is filled by a proxy for children younger than 10 years old and by children themselves when aged ten years and older.

The same CAPTSure© questionnaire is used for self-report and proxy-report, to ease its use in the clinical setting, as it would be difficult to have readily available versions of both self-report and proxy reports for unilateral and bilateral UE and LE questionnaires (8 different versions in total). In its original English version, the wording was developed to accommodate both perspectives, and the translation followed a similar approach.

### Linguistic adaptation

Two professional translators from a commercial translation company, whose native language is French, independently translated the original English version of CAPTSure© for UE and LE symptoms questionnaires and the clinician assessment component. As the translators had no medical background, a pediatric hematologist from the research team served as facilitator. Two professional, non-medical translators, native speakers of English not familiar with the initial index, then performed independent backward translations to identify potential discrepancies from the original tool. An expert committee, including professional translators, research personnel, methodologists and pediatric hematologists from English- and French-speaking backgrounds, reconciled all versions to produce the pre-final French versions of the questionnaires (Additional file 1: Table S1). The reconciliation process took about 2 h, where the translators and the research team carefully discussed all items in sequential order to ensure cross-cultural equivalence.

### Cognitive debriefing interview

To test the pre-final versions of the questionnaires, we piloted the questionnaires and performed cognitive debriefings with semi-structured interviews in the target population of CAPTSure©, that is, children and adolescents at risk of PTS. We enrolled children aged up to 18 years old from the Pediatric Hematology outpatient clinic of the CHU de Québec with a history of UE or LE DVT at least 6 months prior, fluent in French, and who consented to the study. For children who could understand the nature of the study, children’s assent was required. A trained pediatric hematologist performed PTS assessment during a routine clinic visit, and children or their proxies completed the CAPTSure© symptoms assessment.

Cognitive debriefing was conducted by an independent, trained study team member. Cognitive debriefing is a qualitative method to assess respondents’ interpretation of items within a patient-reported outcome. The cognitive debriefing was usually done with children or adolescents, except for young children who were not able to read or had not completed CAPTSure© themselves (in which case the cognitive debriefing was conducted with the parent). The think-aloud technique was used, and patients were asked to explain their thought processes while responding to the questionnaire and probed regarding their answers. Paraphrasing was also used to explore the participant’s comprehension of the questions [12]. Finally, the interviewer asked five general questions to record the global understanding of the questionnaire by the respondents, their appreciation, and any suggestions for improvements. The cognitive debriefing lasted approximately 15 min. Children and adolescents received a gift voucher for their participation. We recorded the debriefings using written notes directly on participants’ questionnaires and using a standardized grid for general comments. Interviews were conducted in a single wave. Following each interview, new data were compared to previously mentioned comments using an iterative process. The problems raised and proposed changes were compiled using an annotated version of the questionnaire and an Excel spreadsheet. Participants were enrolled until reaching saturation (i.e., until no new comments were made after at least two participants in the UE or LE sub-indices).

Finally, once data saturation was felt to be achieved by the study investigator performing the interviews, the study group met to discuss all problems identified by participants and agree on potential solutions, to create the final French versions of the questionnaires. This reconciliation process lasted approximately 1.5 h.

## Results

Following the process of forward and back translations and consensus meetings, 8/14 (57%) of the items in the LE questionnaire and 7/12 (58%) of the items in the UE questionnaire underwent minor wording modifications to preserve the original meaning (Additional file 1: Tables S2 and S3). Pre-final versions were then piloted in twenty respondents, with ten respondents enrolled for both UE and LE sub-indices. Enrolled patients had a median age of 11.5 years old (25th–75th percentile: 6.3–15.0 years) and included 11 males and nine females (Table 1). The median (25th–75th percentile) time in months since DVT diagnosis was 14 (7–16). Twelve patients (60%) had PTS, defined as CAPTSure© > 10, with a median (range) CAPTSure© score of 26 (0–51).

**Table 1:** Characteristics of the study population

Characteristics	Upper extremity DVT (n = 10)	Lower extremity DVT (n = 10)
Age at DVT diagnosis, in years, n (%)		
0–< 5	2 (20)	3 (30)
5–< 10	3 (30)	1 (10)
10–< 15	2 (20)	3 (30)
15–< 18	3 (30)	3 (30)
Age at study enrollment, in years, n (%)		
0–< 5	0	2 (20)
5–< 10	3 (30)	1 (10)
10–< 15	1 (10)	2 (20)
15–< 18	6 (60)	5 (50)
Male sex, n (%)	7 (70)	4 (40)
Location of DVT, n (%)		
Left	4 (40)	4 (40)
Right	6 (60)	6 (60)
Interval since DVT, in months (median, 25–75th percentile)	13 (9–27)	12 (10–27)

*DVT* Deep venous thrombosis

All participants declared the length of the questionnaires to be adequate. While all respondents rated the questionnaires as globally easy to read and comprehend, the participants highlighted some sentences as potentially confusing because they were too long, especially for a respondent with dyslexia. Some pain descriptors were removed, as this vocabulary was not familiar to children and adolescents. Some semantic concerns were also raised. For example, the label “*ma jambe devient enflée*” (becomes swollen) was of concern for children whose leg is perpetually edematous. The label was thus changed to “*ma jambe enfle*” (is swollen). Finally, respondents made comments on the graphical aspects of the questionnaire: the use of cartoons was very appreciated, we modified fonts and colors of some statements that were often looked over by participants, and some text boxes were redesigned to be more visible.

Following the cognitive debriefing, modifications were made to 11/14 (79%) of the items in the LE questionnaire and 9/12 (75%) of the items in the UE questionnaire (Additional file 1: Tables S2 and S3).

Spontaneously, during the semi-structured interviews, a few adolescents expressed overall satisfaction with the use of the questionnaire. One teenage girl voiced her enthusiasm that a questionnaire addressed her specific symptoms: “It feels like I can really explain how I feel.” Another adolescent mentioned: “the form made me realize that I did not voice all of [these findings] to my doctor, because I was not sure if it was related to my clot.”

## Discussion

In this study, we adapted CAPTSure© for French-speaking pediatric patients, using a rigorous methodological process. Despite extensive care put in the initial steps of the process that is, forward translation, backward translation and careful review of all versions by a multidisciplinary team, several additional modifications were made following patients’ input, underlining the importance of field testing for questionnaires, as patients and their families have a unique experience of PTS.

General appreciations’ comments from participants during the process reinforced the usefulness of patient-reported outcomes. The questionnaire was felt not to add a substantial burden on participants, and to improve symptoms’ communication. These comments suggest that using a standardized form like CAPTSure© can improve the detection of patients’ symptoms and increase the clinicians’ understanding of their lived experience. This aligns with other reports supporting that the use of patient-reported outcome measures in clinical practice may improve the detection of disease, enhance patient-provider communication, and increase patient satisfaction with care [13, 14].

Our study has a few limitations. First, our sample size was relatively small, although complying with the methodology suggested by Price and colleagues [8]. However, the same comments were often made by the patients, and no new item was suggested during the final interviews, indicating that we reached data saturation and included the most important modifications. Secondly, Canadian French has its own idioms and colloquialisms. Since this version was mostly tested on French Canadian children and adolescents, this French version might thus need further evaluation before use in other French-speaking jurisdictions. Further work will be performed to validate the translated CAPTSure© in French-speaking patients, to ensure the psychometric properties of its original version are maintained, and to translate CAPTSure© in other languages.

## Conclusions

In conclusion, we have translated and adapted CAPTSure© in French. This will ease the diagnosis and severity rating of PTS in children in clinical practice and allow international research collaborations for non-English-speaking patients. Interested readers may contact pts.research@sickkids.ca for potential licensing of the tool at their institutions.

### Supplementary Information

**Additional file 1: Table S1.** Multidisciplinary study team (in alphabetical order). **Table S2.** Summary of comments and examples made to Lower Extremity Questionnaire. **Table S3.** Summary of comments and examples made to Upper Extremity Questionnaire.

## Data Availability

The data that support the findings of this study are available from the corresponding author, but restrictions apply to the availability of these data due to privacy protection and ethical regulations and so are not publicly available. Data are however available from the authors upon reasonable request following appropriate ethical review.

## Abbreviations

CAPTSure©: Index for the Clinical Assessment of Post-Thrombotic Syndrome in children
CHU: Centre Hospitalier Universitaire
DVT: Deep venous thrombosis
LE: Lower extremity
PTS: Post-thrombotic syndrome
UE: Upper extremity
VTE: Venous thromboembolism
