# Supplementary material for: French Canadian cross-cultural adaptation of CAPTSure©, an index for the Clinical Assessment of Post-Thrombotic Syndrome in children
Source: J Patient Rep Outcomes. 2023 Aug 18;7:83. doi: 10.1186/s41687-023-00622-7 (PMC10439081; doi:10.1186/s41687-023-00622-7)
Supplement: Supplementary file 1 — Additional file 1: Table S1. Multidisciplinary study team (in alphabetical order). Table S2. Summary of comments and examples made to Lower Extremity Questionnaire. Table S3. Summary of comments and examples made to Upper Extremity Questionnaire. [file 41687_2023_622_MOESM1_ESM.docx]

**Supplementary Table 1. Multidisciplinary study team (in alphabetical order)**

| **Study team member** | **Institution** | **Expertise/role** |
| --- | --- | --- |
| L Avila | The Hospital for Sick Children | Principal investigator for original CAPTSure© development  Clinician-scientist with expertise in pediatric thrombosis and clinical epidemiology |
| Translators (4), including a senior project manager | Canadian commercial translation company | Independent translation of the questionnaire, from French to English and English to French |
| MC Pelland-Marcotte | Centre Hospitalier Universitaire de Québec | Clinician-scientist with expertise in pediatric thrombosis and clinical epidemiology, whose native language in French |
| R Santiago | Centre Hospitalier Universitaire de Québec | Clinician in pediatric hematology |
| Research personnel | The Hospital for Sick Children | Coordination of tasks and editorial support |

**Supplementary Table 2. Summary of comments and examples made to Lower Extremity Questionnaire**

| **Participants’ comments** | **Items affected** | **Example of Change*** | | |
| --- | --- | --- | --- | --- |
|  |  | **Initial statement** | **Changes following back translations** | **Changes following cognitive debriefings** |
| Unnecessarily wordy (n=3) | 1, 2, 5 | (…) combien de fois (…) eu l’un des problèmes suivants dans la jambe où se trouvait le caillot par rapport à l’autre jambe? | Typo identified | (…) combien de fois (…) eu l’un des problèmes suivants? |
| Incorrectly worded (n=3) | 1c, 1d, 6b | Par rapport (…) devenait enflée. | (…) devient enflée. | (…) est enflée. |
| « Funny» sounding (n=2) | 1f, 3 | Les problèmes de jambe que vous avez sélectionnés à la question 1 sont-ils plus graves pendant (…) | « Exercice » changed for broader « activité physique » | Les problèmes de jambe sélectionnés à la question 1 sont-ils pires pendant (…) |
| Hard to Understand (n=3) | 6a, 6c, 7 | Encercle tous les mots qui décrivent ta douleur | Felt to be hard to translate | Complete list of pain descriptors added with problematic or redundant descriptors removed |

* The full list of changes is not provided due to copyright issues. Please reach out to the corresponding author for each specific change made to address these comments.

**Supplementary Table 3. Summary of comments and examples made to Upper Extremity Questionnaire**

| **Participants’ comments** | **Items affected** | **Example of Change*** | | |
| --- | --- | --- | --- | --- |
|  |  | **Initial statement** | **Changes following back translations** | **Changes following cognitive debriefings** |
| Unnecessarily wordy (n=3) | 1c, 2, 5 | (…) causait des picotements. | (…) cause des picotements. | (…) picote. |
| Incorrectly worded (n=2) | 1b, 6b | (…) à quelle fréquence as-tu eu de la douleur (…)? | N/A | (…) à quelle fréquence as-tu ressenti de la douleur (…)? |
| « Funny» sounding (n=1) | 3 | Les problèmes de bras que vous avez sélectionnés à la question 1 sont-ils plus graves pendant (…) | « Exercice » changed for broader « activité physique »  e. | Les problèmes de bras sélectionnés à la question 1 sont-ils pires pendant (…) |
| Hard to Understand (n=3) | 1, 6c, 7 | Encercle tous les mots qui décrivent ta douleur | Felt to be hard to translate | Complete list of pain descriptors added with problematic or redundant descriptors removed |

* The full list of changes is not provided due to copyright issues. Please reach out to the corresponding author for each specific change made to address these comments.
